# CHL1 gene polymorphisms increase lung cancer susceptibility

**DOI:** 10.18632/oncotarget.24057

**Published:** 2018-01-06

**Authors:** Wen Tian, Xuelian Li, Yangwu Ren, Zhihua Yin, Xiaowei Quan, Chang Zheng, Baosen Zhou

**Affiliations:** ^1^ Department of Epidemiology, School of Public Health, China Medical University, Shenyang, China; ^2^ Key Laboratory of Cancer Etiology and Prevention, China Medical University, Liaoning Provincial Department of Education, Liaoning, Shenyang, China

**Keywords:** polymorphism, lung cancer, susceptibility, CHL1

## Abstract

Lung cancer represents a complex and malignant cancer. Close Homologue of L1 (CHL1) gene plays a crucial role in the progress of cancer. The aim of this study is to explore the association between CHL1 rs425366 polymorphism and lung cancer susceptibility in northeast of China. A hospital-based case-control study was carried out to collect relative characteristics. Logistic regression analysis was conducted to analyze the relationship between single nucleotide polymorphisms and lung cancer susceptibility. The results suggested that there was statistically significant difference between GT genotype and TT genotype of rs425366 and lung cancer susceptibility. In stratified analysis, TT genotype of rs425366 may increase the risk of lung adenocarcinoma. We also found that non-smoking individuals carrying T allele were more likely to develop lung cancer. Overall, our study may indicate that CHL1 gene may increase lung cancer susceptibility in northeast of China.

## INTRODUCTION

Lung cancer is one of the complicated and malignant diseases, causing 1.8 million new cases and 1.6 million deaths annually in the worldwide [1–3]. In China, the increase of lung cancer hits 1.63% per year, 1.30% for men and 2.34% for women [4, 5]. In spite of significant breakthrough in the treatment of lung cancer, 5-year survival rate still remains less than 10% [6, 7]. As the research moved along, tobacco exposure was considered as one of the potential risk factors for lung cancer [8-11]. However, there are still a large proportion of non-smoking patients suffering from lung cancer in their lifetime [12]. Thus, gene polymorphisms and environmental factors play prominent roles in the process of cancer occurrence, progression and treatment [13, 14].

CHL1 gene, on 3p26, is a neural recognition molecule which may be involved in signal transduction pathways [15, 16]. The protein encoded by CHL1 gene belongs to L1 gene family of neural cell adhesion molecules [17, 18]. For cervical cancer cells, CHL1 gene promoted cell growth, migration and invasion as a target of miR-10a [19]. In human breast cancer cells, the deficiency of CHL1 gene may dysregulate of MAPK and PKA pathways. Finally CHL1 gene promoted cancer cells growth, migration and invasion [20–22]. Other data testified that abnormal expression of CHL1 gene inhibited cancer cells clonogenicity and migration in nasopharyngeal carcinoma [23]. Meanwhile, recent research showed that the up-regulation of CHL1 gene and increase of CHL1 mRNA level were found in lung cancer [17]. Until now, the biological mechanism of CHL1 gene in lung cancer is still an issue. Thus, we investigated the biological function of CHL1 gene.

In this paper, we conducted hospital-based case-control study to explore the relationship between CHL1 gene polymorphism and lung cancer susceptibility. Besides, we investigated the association between CHL1 gene polymorphism, tobacco exposure and lung cancer susceptibility.

## RESULTS

### Subjects characteristics

This study consisted of 742 cases and 679 controls, involving 174 smokers and 1005 non-smokers. As Table 1 showed, the mean ages of cases and controls were 58.06 ± 11.306 and 57.98 ± 10.625 years, respectively. The results pointed out that there were no significant differences in gender, age and smoking status between cases and controls (Gender: *P* = 0.231; Age: *P* = 0.320; Smoke: *P* = 0.936). Lung cancer patients were consisted of 454 adenocarcinomas, 164 squamous cell carcinomas, 92 small cell adenocarcinomas.

**Table 1:** Demographic characteristics of cases and controls in northeast of China

Variables		Cases (%)	Controls (%)	*P*-value
Gender	male	131 (17.66)	129 (19.00)	
	female	611 (82.34)	550 (81.00)	0.231
Age (years)	mean ± SD	58.06 ± 11.306	57.98 ± 10.625	0.320
Smoke	never	627 (84.39)	378 (55.67)	
	yes	108 (14.54)	66 (9.72)	0.936
	unknown	8 (1.07)	235 (34.61)	
Histology	adenocarcinoma	454 (61.19)		
	squamous cell carcinoma	164 (22.10)		
	small cell lung cancer	92 (12.40)		
	others	32 (4.31)		

### SNP frequencies and association with lung cancer

Table 2 tabulated distribution of rs425366 G > T in cases and controls. The genotype frequencies of rs425366 polymorphism in controls were in accordance with Hardy-Weinberg equilibrium (*P* = 0.958). After adjusting by age and gender, we found statistically significant association of rs425366 polymorphism and lung cancer susceptibility. The result explored that carriers of T allele had an increased risk of lung cancer compared with homozygous wild allele (T allele: *P* = 0.018). Moreover, TT genotype of rs425366 had a 1.433-fold risk of lung cancer susceptibility compared with GT genotype (TT: *P* = 0.019, OR = 1.433; GT: *P* = 0.031, OR = 1.310).

**Table 2:** Distribution of CHL1 rs425366 and the association with lung cancer susceptibility for cases and controls in northeast of China

	Genotype	Case	Control	OR (95%CI) ^*^	*P*-value	HWD
rs425366	GG	188 (25.34)	213 (31.37)	Ref		0.958
	GT	388 (52.29)	334 (49.19)	1.310 (1.025, 1.674)	0.031	
	TT	166 (22.37)	132 (19.44)	1.433 (1.060, 1.938)	0.019	
	Dominant model			1.343 (1.065, 1.694)	0.013	
	Recessive model			1.194 (0.924, 1.544)	0.175	
	Allele model			1.196 (1.032, 1.387)	0.018	

^*^Adjusted by for age and gender, OR and 95% CI were calculated by logistic regression.

### Stratified analysis

For rs425366 polymorphism, we found statistically significant association between TT genotype and lung adenocarcinoma (TT: *P* = 0.026, OR = 1.477). In addition, individuals carrying T allele of CHL1 gene had a 1.207-fold risk of lung adenocarcinoma (Additive model: *P* = 0.030). Nevertheless, we failed to observe significant differences between squamous cell carcinoma and gene polymorphism (GT: *P* = 0.257, OR = 1.271; TT: *P* = 0.104, OR = 1.515; Dominant model: *P* = 0.145; OR = 1.340; Additive model: *P* = 0.107, OR = 1.224).The similar results were also found in small cell lung cancer (GT: *P* = 0.265, OR = 1.352; TT: *P* = 0.990, OR = 1.004; Dominant model: *P* = 0.533; OR = 1.168; Additive model: *P* = 0.981, OR = 0.996).

### Gene-environmental analysis

Table 4 described the effect of gene-environment on lung cancer susceptibility in northeast of China. The results indicated that non-smoking individuals who carried rs425366 T allele had a 1.248-fold increased risk for developing lung cancer (*P* = 0.018). No significant association was observed between tobacco exposure, rs425366 polymorphism and lung cancer susceptibility.

**Table 3:** Stratified analysis of CHL1 rs425366 and lung cancer susceptibility in northeast of China

Histology	Genotype	Case	Control	OR (95% CI)^*^	*P*-value
adenocarcinoma	GG	113 (24.89)	213 (31.37)	Ref	
	GT	236 (51.98)	334 (49.19)	1.293 (0.973, 1.720)	0.077
	TT	105 (23.13)	132 (19.44)	1.477 (1.047, 2.084)	0.026
	Dominant model			1.336 (1.021, 1.750)	0.035
	Recessive model			1.234 (0.922, 1.652)	0.157
	Allele model			1.207 (1.018, 1.430)	0.030
squamous cell carcinoma	GG	42 (25.61)	213 (31.37)	Ref	
	GT	83 (50.61)	334 (49.19)	1.271 (0.840, 1.925)	0.257
	TT	39 (23.78)	132 (19.44)	1.515 (0.918, 2.503)	0.104
	Dominant model			1.340 (0.904, 1.985)	0.145
	Recessive model			1.274 (0.842, 1.928)	0.252
	Allele model			1.224 (0.957, 1.565)	0.107
small cell lung cancer	GG	26 (28.26)	213 (31.37)	Ref	
	GT	51 (55.43)	334 (49.19)	1.258 (0.757, 2.092)	0.375
	TT	15 (16.31)	132 (19.44)	0.950 (0.478, 1.888)	0.883
	Dominant model			1.168 (0.717, 1.903)	0.533
	Recessive model			0.792 (0.439, 1.430)	0.440
	Allele model			0.996 (0.728, 1.364)	0.981

^*^Adjusted by age and gender, OR and 95% CI were calculated by logistic regression

**Table 4:** The effect of CHL1 rs425366 on lung cancer susceptibility in smokers and non-smokers in northeast of China

		Genotype	Case	Control	OR (95% CI) ^*^	*P*-value
smoking	yes	GG	30 (27.78)	144 (32.43)	Ref	
		GT	50 (46.30)	216 (48.65)	1.035 (0.532, 2.017)	0.918
		TT	28 (25.92)	84 (18.92)	1.797 (0.805, 4.011)	0.153
		Dominant model			1.227 (0.660, 2.282)	0.518
		Recessive model			1.775 (0.896, 3.520)	0.100
		Allele model			1.333 (0.890, 1.995)	0.163
	no	GG	156 (24.88)	121 (32.10)	Ref	
		GT	335 (53.43)	184 (48.81)	1.514 (1.119, 2.049)	0.007
		TT	136 (21.69)	72 (19.09)	1.538 (1.056, 2.242)	0.025
		Dominant model			1.522 (1.142, 2.027)	0.004
		Recessive model			1.175 (0.852, 1.622)	0.326
		Allele model			1.248 (1.039, 1.499)	0.018

^*^Adjusted by age and gender, OR and 95% CI were calculated by logistic regression

## DISCUSSION

Gene polymorphism and environmental factors play key roles in lung cancer initiation and progression. We performed this case-control study to estimate the connection between CHL1 gene polymorphism and lung cancer susceptibility. As far as we are aware, this paper firstly investigated the connection between CHL1 gene polymorphism and lung cancer susceptibility. Our results showed that there was statistically significant association between CHL1 rs425366 polymorphism and lung cancer susceptibility in northeast of China. In addition, T allele of rs425366 in CHL1 gene increased lung cancer susceptibility.

rs425366 locates in the intron zone of CHL1 gene, which may not alter the sequence of mRNA and protein [23]. But mutations in rs425366 may modulate CHL1 gene biological function in different human cancer cell lines [23]. CHL1 gene is located at 3p26, which is demonstrated to be a candidate for prostate cancer susceptibility in Finnish prostate cancer families [17]. In review of previous researches, biological role and mechanism of CHL1 gene are issues in different human cancers. In ovarian cancer, Emily et al. proposed that CHL1 gene could not be identified as the tumor suppressor gene because somatic mutations of the gene were not identified [24]. On the other side, Sencheoko et al. investigated that CHL1 gene was a putative tumor suppressor in growth of ovarian tumor [17]. Meanwhile, because of the loss of 3p26.3, CHL1 gene was also reported as a candidate tumor suppressor gene in oral squamous cell carcinoma and esophageal squamous cell carcinoma [25, 26]. Genome Wide Association Study (GWAS) had found that CHL1 gene polymorphism had a link with lung cancer. In previous study, Vera N. Senchenko et al. demonstrated that CHL1 expression and CHL1 mRNA both decreased in lung cancer [17]. Moreover, the frequency of CHL1 mRNA decrease was different in different histology [17]. This controversial conclusion might be related to different ethnic, human cancers and life habits.

In this paper, we explored that there was a significant positive correlation between rs425366 polymorphism and lung cancer susceptibility in northeast of China. Additionally, individuals carrying at least one T allele (GT/TT) were less likely to suffer from lung cancer, comparing with individuals carrying GG genotype.

Furthermore, the stratified analysis results showed that T allele of rs425366 had a 1.477-fold risk of lung adenocarcinoma. However, no statistical associations were observed between GT genotype and lung adenocarcinoma. There was no evident correlation between lung squamous cell carcinoma, small cell lung cancer and gene polymorphism. These conflicting results might be due to the limited sample sizes. Thus, larger population is warranted to confirm the results in the future.

Tobacco carcinogens in smoking could result in gene mutations [27]. Individuals who exposed to tobacco trend to develop lung cancer [28]. Finally, we dissected the effect of gene-environment in lung cancer. However, our results indicated that non-smoking individuals carrying rs425366 GT and TT genotypes may have more risk of lung cancer. There was no statistically significant effect between tobacco exposure and gene polymorphism in lung cancer susceptibility. There was no significant interaction between rs425366 polymorphism and tobacco exposure in northeast of China (The result was not shown; *P* = 0.140). The impact of CHL1 gene polymorphism may modulate the function of tobacco exposure. Hence we need a larger sample for further study to manifest the mechanism of CHL1 gene.

However, there are several limitations in our present study. Firstly, the number of our sample involved is not large enough. We only obtained demographic information of northeast of China. Thus, larger sample sizes are necessary in the further research. Secondly, we adopted hospital-based study, which may lead to selection bias. Thirdly, we lacked other gene-environmental interaction to verify the connection between CHL1 gene and lung cancer. Hence rigorous research will be needed to clarify the speculation about CHL1 gene and lung cancer.

In conclusion, we demonstrated that there was a link between CHL1 gene and lung cancer susceptibility in northeast of China. Moreover, this study manifested that rs425366 in CHL1 gene may increase the risk of lung adenocarcinoma. However, the results should be verified in a larger population. In the future study, it will be especially necessary to investigate the biological function of CHL1 gene in lung cancer.

## MATERIALS AND METHODS

### Subject data collection

This hospital-based case-control study was performed in Shenyang, containing 742 cases and 679 controls. All patients were recruited from the First Affiliated Hospital of China Medical University, the Fourth Affiliated Hospital of China Medical University and Liaoning Cancer Hospital. The cases were histologically confirmed lung cancer between March 2010 and March 2013. The controls were frequency-matched and recruited from the same hospitals. The questionnaires were set to gather specific demographic characteristics including age, gender, smoking status, pathologic type, etc. Subjects who had smoked more than 100 cigarettes in the lifetime were defined as smokers. Those who had smoked less than 100 cigarettes were defined as non-smokers. All studies were conformed by the local institutional review board and all involved patients signed informed consent agreement.

### DNA genotyping

In this study, genomic DNA samples of all subjects were segregated by phenol chloroform methods. Taqman allelic discrimination assay was used to estimate SNP genotyping. PCR Taqman primers and probes were supplied by Applied Biosystems. The results were obtained by ABI 7500 Fast Real-time PCR System with the Sequence Detection Software. Gene magnification was heated at 95°C for 10 min, 53 cycles at 92°C for 30s and 60°C for 1 min. To maintain accuracy of analysis results, ten percent of samples were duplicated and the results were coherent.

### Statistical analysis

The two sides χ^2^ test and *t* test were conducted to examined the characteristics of cases and controls. Logistic regression model was used to evaluate the odd ratios (ORs) and 95% confidence intervals (CIs). *P* < 0.05 was defined as statistical significance. Hardy-Weinberg equilibrium for SNP was calculated by the goodness-of-fit χ^2^ test. SPSS 20.0 software was utilized to analyze all data.
